# Methemoglobin Is an Endogenous Toll-Like Receptor 4 Ligand—Relevance to Subarachnoid Hemorrhage

**DOI:** 10.3390/ijms16035028

**Published:** 2015-03-05

**Authors:** Min Seong Kwon, Seung Kyoon Woo, David B. Kurland, Sung Hwan Yoon, Andre F. Palmer, Uddyalok Banerjee, Sana Iqbal, Svetlana Ivanova, Volodymyr Gerzanich, J. Marc Simard

**Affiliations:** 1 Department of Neurosurgery, University of Maryland School of Medicine, Baltimore, MD 21201, USA; E-Mails: mskwon@smail.umaryland.edu (M.S.K.); skwoo@smail.umaryland.edu (S.K.W.); kurland.davidb@gmail.com (D.B.K.); sana85nov@hotmail.com (S.Iq.); sveta0652@yahoo.com (S.Iv.); vgerzanich@smail.umaryland.edu (V.G.); 2 Department of Pathology, University of Maryland School of Medicine, Baltimore, MD 21201, USA; 3 Department of Physiology, University of Maryland School of Medicine, Baltimore, MD 21201, USA; 4 Department of Pharmaceutical Sciences, University of Maryland School of Pharmacy, Baltimore, MD 21201, USA; E-Mail: syoon@rx.umaryland.edu; 5 William G. Lowrie Department of Chemical and Biomolecular Engineering, the Ohio State University, Columbus, OH 43210, USA; E-Mails: palmer.351@osu.edu (A.F.P.); banerjee.49@osu.edu (U.B.)

**Keywords:** subarachnoid hemorrhage, methemoglobin, hemoglobin, Toll-like receptor 4, tumor necrosis factor α, neuroinflammation

## Abstract

Neuroinflammation is a well-recognized consequence of subarachnoid hemorrhage (SAH), and may be responsible for important complications of SAH. Signaling by Toll-like receptor 4 (TLR4)-mediated nuclear factor κB (NFκB) in microglia plays a critical role in neuronal damage after SAH. Three molecules derived from erythrocyte breakdown have been postulated to be endogenous TLR4 ligands: methemoglobin (metHgb), heme and hemin. However, poor water solubility of heme and hemin, and lipopolysaccharide (LPS) contamination have confounded our understanding of these molecules as endogenous TLR4 ligands. We used a 5-step process to obtain highly purified LPS-free metHgb, as confirmed by Fourier Transform Ion Cyclotron Resonance mass spectrometry and by the Limulus amebocyte lysate assay. Using this preparation, we show that metHgb is a TLR4 ligand at physiologically relevant concentrations. metHgb caused time- and dose-dependent secretion of the proinflammatory cytokine, tumor necrosis factor α (TNFα), from microglial and macrophage cell lines, with secretion inhibited by siRNA directed against TLR4, by the TLR4-specific inhibitors, Rs-LPS and TAK-242, and by anti-CD14 antibodies. Injection of purified LPS-free metHgb into the rat subarachnoid space induced microglial activation and TNFα upregulation. Together, our findings support the hypothesis that, following SAH, metHgb in the subarachnoid space can promote widespread TLR4-mediated neuroinflammation.

## 1. Introduction

Aneurysmal subarachnoid hemorrhage (SAH) can be devastating. Overall, nearly 50% of patients die, and those who survive often have poor long-term outcomes. Surviving patients are at risk for potentially severe complications, including vasospasm, cognitive dysfunction and global brain atrophy [1]. Cognitive dysfunction afflicts up to half of survivors, manifests as deficits in memory, executive function and language [2,3,4,5], and is responsible for the fact that only a third of previously employed SAH survivors return to work [6]. Cognitive dysfunction may be linked to global brain atrophy, which is very common in SAH, is strongly associated with poor outcome [7,8], and correlates with the severity of the systemic inflammatory response [9]. Brain atrophy has been linked to persistent inflammation in other conditions as well [10,11,12,13].

Toll-like receptors (TLRs) comprise a family of 13 distinct receptors that play key roles in innate immunity. Among them, TLR2 and TLR4, which are widely expressed in the brain [14], can detect endogenous ligands called alarmins or danger associated molecular patterns (DAMPs). To date, more than 20 endogenous TLR ligands have been proposed that may be categorized as follows: released intracellular proteins, extracellular matrix components, oxidatively modified lipids, and other soluble mediators [15]. TLR signaling via the pathway involving myeloid differentiation primary response gene 88 (MyD88) culminates in the activation of nuclear factor κB (NFκB) and of mitogen-activated protein kinases. Notably, persistent activation of TLR4 in the brain is one of the few molecular mechanisms established as a cause of long-lasting cognitive dysfunction [16,17,18], with cognitive dysfunction being tumor necrosis factor α (TNFα)-dependent [19,20].

Signaling by TLR4-mediated NFκB is increasingly recognized to play a crucial role in the pathogenesis of neuronal damage after SAH [21,22,23,24]. TLR4 mRNA and protein are upregulated in the brains and blood vessels of SAH patients and of animal models of SAH [21,22,25]. In a rabbit model, increased TLR4 expression correlates with the appearance of cerebral vasospasm [22], and treatment with the peroxisome proliferator-activated receptor (PPAR) γ agonist, rosiglitazone, inhibits both TLR4 upregulation and vasospasm [26]. Recent work based on gene knockout mice has confirmed the critical role of TLR4 and the MyD88 pathway, especially in microglia, in SAH-induced vasospasm and neuronal cell death [24].

Erythrocyte hemolysate, some components of which mimic adverse consequences of SAH [27], contains three purported endogenous TLR4 ligands: methemoglobin (metHgb[Fe^3+^]) [28,29], heme [30,31] and hemin [32]. metHgb can activate either TLR4 dimers or TLR4/TLR2 heterodimers [28,29]. Although oxyHgb[Fe^2+^] has been claimed to activate TLR4 in cultured vascular smooth muscle cells [33], it is likely that spontaneous oxidation under normal culture conditions would have converted Hgb to metHgb. Unlike heme and hemin, which are sparingly soluble in water, and which are rapidly degraded by heme oxygenase-1 [34], metHgb is abundantly soluble in water, allowing it to be widely dispersed by CSF hydrodynamics, and thereby induce potentially widespread TLR4 activation throughout the brain. Notably, levels of metHgb following intraventricular hemorrhage correlate directly with levels of the proinflammatory cytokine, TNFα [35], a key downstream product of TLR4 activation [19,20]. In SAH, as blood ages, metHgb becomes the dominant form of hemoglobin present in the subarachnoid space [36]. For these reasons, metHgb is highly attractive as the candidate TLR4 ligand that could be responsible for many of the global effects of SAH. 

Work on endogenous TLR4 ligands has been hampered by potential contamination by the classic ligand, lipopolysaccharide (LPS). LPS is a highly potent TLR4 ligand, with 100–200 pg/mL sufficient to maximally induce TNFα release from macrophages [37]. Some commercial preparations of metHgb have been reported to contain as much as 400 pg LPS per mg Hgb [28], although others reportedly contain much less (<5 pg/mg Hgb [38]). Considerable effort is required to prepare highly purified, LPS-free Hgb [39]. The two studies that reported metHgb activation of TLR4 used commercially available hemolysate as is, with experiments performed in the presence of polymyxin B [28,29], a strategy commonly employed to inactivate LPS contaminants [15,40]. Nevertheless, numerous examples exist demonstrating how LPS contamination of candidate TLR4 ligands can lead to mistaken conclusions [40,41]. Indeed, in a recent review of 23 reported endogenous ligands of TLR2 and/or TLR4, fully 8 were shown to have no cytokine stimulatory effects when highly purified preparations were used [15]. Great caution is required when studying TLR4 signaling relevant to SAH.

Here, we used a 5-step process to obtain highly purified LPS-free metHgb from hemolysate. We show that metHgb is a TLR4 ligand at physiologically relevant concentrations, that metHgb induces the secretion of TNFα in a time- and dose-dependent manner from microglial and macrophage cell lines, and that metHgb injection into the rat subarachnoid space results in robust microglial activation.

## 2. Results and Discussion

### 2.1. Purification of Bovine metHgb

**Figure 1:**
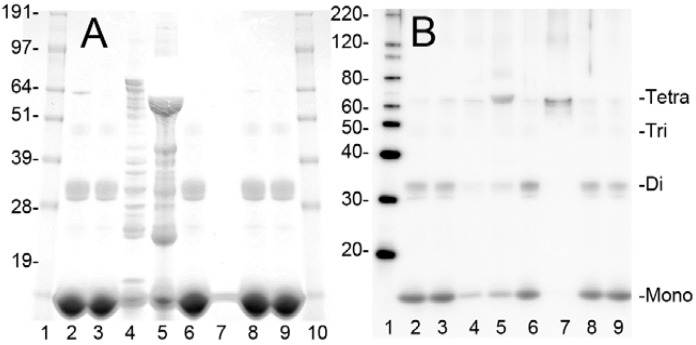
Purification of bovine metHgb. (**A**,**B**) ~50 or ~5 µg of protein from individual steps during purification of bovine metHgb were examined by SDS-PAGE with Coomassie G-250 staining (**A**) and immunoblot analysis for hemoglobin α subunit (**B**). Samples include starting hemolysate (lane 2), flow-through fraction from Q-Sepharose (lane 3), eluate from Q-Sepharose with 0.1 M NaCl (lane 4) and with 0.5 M NaCl (lane 5), fraction from ultrafiltration (lane 6), flow-through fraction of the ultrafiltration (lane 7), fractions from PD-10 (lane 8), and from EndoTrap HD (lane 9). Molecular masses for marker proteins are shown (lanes 1 and 10 for **A**; lane 1 for **B**). Monomeric hemoglobin α and β subunits (Mono) and hemoglobin dimers (Di), trimers (Tri), and tetramers (Tetra) are marked.

The steps used to obtain purified LPS-free metHgb from hemolysate, and the yield at each step, are summarized in Table 1. Fractions from each step were examined by sodium dodecyl sulfate polyacrylamide gel electrophoresis (SDS-PAGE) with Coomassie G-250 staining and immunoblot analysis of the hemoglobin α subunit (Figure 1). SDS-PAGE and immunoblot analyses revealed that the hemolysate contained hemoglobin along with many other proteins. Hemoglobin α and β chains migrated as a 15 kDa band, as expected (Figure 1A,B). Two additional bands, with estimated molecular masses of 32 and 64 kDa, also were detected by SDS-PAGE, with immunoblot indicating that these bands represented hemoglobin dimers and tetramers, respectively [39]. Most of the protein, including metHgb, did not bind to the Q-Sepharose column and was collected as flow-through. However, many hemolysate proteins did bind to the column; about 2% of the loaded protein, including numerous non-hemoglobin proteins, was recovered from the Q-Sepharose column by elution with high concentrations of NaCl (Figure 1 A,B, lanes 4 and 5). The flow-through fraction from the Q-Sepharose column was concentrated using an ultrafiltration unit with nominal molecular mass limit of 30 kDa. Less than 0.05% of the protein passed through the ultrafiltration unit membrane, indicating that most of the metHgb maintained its multimeric structure during the initial preparation steps. Impurities with small molecular mass such as free heme were removed by gel filtration column chromatography with an exclusion limit of 5 kDa (PD-10). Lastly, LPS was removed using LPS-removal column chromatography (EndoTrap HD). A Limulus amebocyte lysate (LAL) assay detected no LPS in the purified metHgb preparation, whereas the initial hemolysate before purification was found to contain ~10 endotoxin units/mg of protein. The typical protein yield from this multi-step process was ~65%.

**Table 1:** Purification of bovine hemoglobin.

Steps	Protein (mg)	Yield (%)
Starting hemolysate	2094	100
Centrifugation	2017	96
Q-Sepharose	1822	87
Ultrafiltration	1651	79
PD-10	1374	66
EndoTrap HD	1364	65

**Figure 2:**
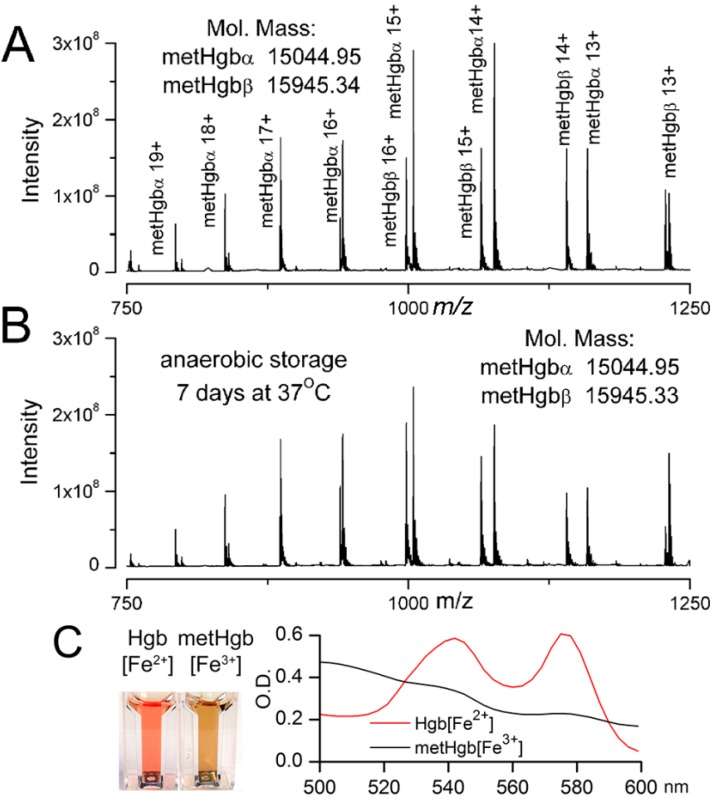
Characterization of metHgb after purification. (**A**,**B**) FT-ICR mass spectrometry of purified LPS-free metHgb that was freshly prepared (**A**); and of purified LPS-free metHgb maintained in solution under anaerobic conditions at 37 °C for 7 days (**B**), showing families of peaks for multiprotonated metHgb α or β and no contaminants; *m*/*z*, mass divided by charge; deconvoluted molecular masses for singly protonated hemoglobin α and β subunits, determined from the mass spectrum, also are shown; (**C**) Images (**left** panel) and visible spectra (**right** panel) of Hgb[Fe^2+^] and metHgb[Fe^3+^] solutions.

To measure the molecular mass of the purified metHgb and to detect any potential contaminants, the purified metHgb sample was examined using a 12 Tesla Fourier Transform Ion Cyclotron Resonance (FT-ICR) mass spectrometer. Heme (616.1767 Da) was used as an internal mass calibrant. The mass spectrum revealed a family of peaks of multiply charged hemoglobin α and β subunits (Figure 2A). The molecular masses for singly protonated hemoglobin α and β subunits were determined to be 15,044.9476 and 15,945.3381 Da, respectively, which are consistent with their theoretical values: 15,044.9115 Da for α and 15,945.3175 Da for β subunits. Consistent with previous reports [39], hemoglobin α subunits lacked the *N*-terminal methionine residue. No other molecules were detected by FT-ICR mass spectrometry, confirming that the metHgb sample was ultra-pure.

We also examined the stability of purified LPS-free metHgb maintained in solution under anaerobic conditions at 37 °C for 7 days. FT-ICR mass spectrometry of metHgb stored under these conditions revealed a spectrum identical to that of freshly prepared metHgb with no degradation products (Figure 2B), confirming that purified metHgb was free of proteolytic activity and was stable.

Spectrophotometry confirmed that purified LPS-free hemoglobin prepared as described was predominantly the oxidized form, metHgb[Fe^3+^], not the reduced form, Hgb[Fe^2+^] (Figure 2C). Except where otherwise indicated, we used this highly purified, LPS-free metHgb in all subsequent experiments.

### 2.2. metHgb Induces p65 Nuclear Translocation in Microglia

**Figure 3:**
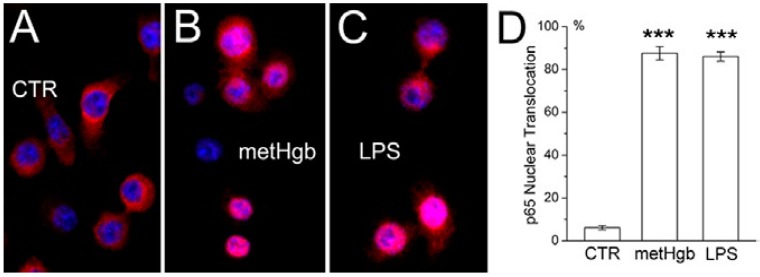
metHgb causes NFκB activation in microglia. (**A**–**C**) Cultured microglia under control conditions (**A**); or after 60-min exposure to purified LPS-free metHgb (7 mg/mL) (**B**) or LPS (100 pg/mL) (**C**), immunolabeled with anti-p65 antibody; nuclei labeled with DAPI (blue); pink nuclei indicate nuclear translocation of p65; (**D**) Percent of nuclei showing translocation of p65 1 h after exposure to purified LPS-free metHgb (7 mg/mL) or LPS (100 pg/mL); 3 replicates per treatment; ***, *p* < 0.001.

TLR4 ligation results in activation of NFκB [42,43]. Exposure of cultured microglia to metHgb resulted in nuclear translocation of p65, which was evident at 60 min, and was similar to that induced by the classic TLR4 ligand, LPS (Figure 3A–C). At a concentration 20 times less than that in blood, metHgb (7 mg/dL) was as efficacious as LPS (100 pg/mL) at inducing p65 nuclear translocation (Figure 3D).

### 2.3. metHgb Induces Secretion of TNFα in Microglia and Macrophages

**Figure 4:**
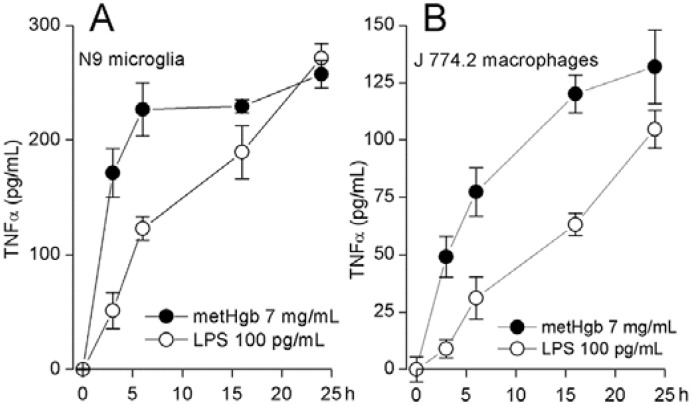
metHgb causes time-dependent secretion of TNFα from microglia and macrophages. (**A**,**B**) Cultured N9 microglia (**A**) and J 774.2 macrophages (**B**) were exposed to purified LPS-free metHgb (7 mg/mL) or LPS (100 pg/mL), as indicated; TNFα secretion into the medium was measured at different times by ELISA; 3 replicates per condition.

**Figure 5:**
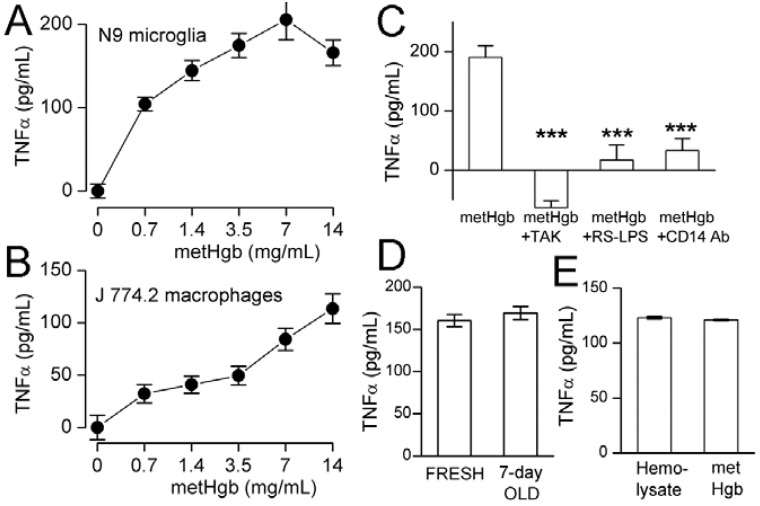
metHgb causes dose-dependent, TLR4-dependent secretion of TNFα from microglia and macrophages. (**A**,**B**) Cultured N9 microglia (**A**) and J 774.2 macrophages (**B**) were exposed to purified LPS-free metHgb at the concentrations indicated on the abscissae; (**C**) Cultured N9 microglia were exposed to purified LPS-free metHgb (7 mg/mL) without or with TAK-242 (2 µM), or Rs-LPS (10 µM), or anti-CD14 antibody (10–50 µg/mL), as indicated; (**D**) Cultured N9 microglia were exposed to purified LPS-free metHgb (7 mg/mL), either freshly prepared or stored in solution under anaerobic conditions at 37 °C for 7 days; (**E**) Cultured N9 microglia were exposed to LPS-free hemolysate (7 mg/mL) or purified LPS-free metHgb (7 mg/mL). In all cases (**A**–**E**), TNFα secretion into the medium was measured at 24 h by ELISA; for (**A**,**B**,**D**,**E**), 3 replicates per condition; for (**C**), 4–8 replicates per condition; *** *p* < 0.001.

TLR4 ligation causes TNFα secretion by macrophages [44] and microglia [45,46]. metHgb caused time- and dose-dependent secretion of TNFα from both microglial and macrophage cell lines (Figure 4A,B and Figure 5A,B). With both microglia and macrophages, TNFα secretion was detected as early as 3 h after exposure to metHgb (7 mg/mL) (Figure 4A,B). Whereas TNFα secretion reached a plateau by about 5 h in microglia (Figure 4A), it continued to rise over the course of 24 h in macrophages (Figure 4B). In both microglia and macrophages, the time-courses of TNFα secretion induced by metHgb appeared to be slightly faster than the time-courses observed with LPS (100 pg/mL) (Figure 4A,B).

### 2.4. metHgb-Induced TNFα Secretion Is TLR4-Dependent

TAK-242 (resatorvid) is a specific small-molecule inhibitor of TLR4 signaling that selectively binds to Cys747 on the intracellular domain of TLR4 and interrupts interactions between TLR4 and its adaptor molecules [47]. We used TAK-242 to evaluate the role of TLR4 in metHgb-induced secretion of TNFα [31]. In microglia, TAK-242 completely eliminated metHgb-induced TNFα secretion, as well as baseline, unstimulated secretion of TNFα (Figure 5C). TAK-242 also was highly effective at inhibiting TNFα secretion from macrophages (not shown).

The role of TLR4 in metHgb-induced TNFα secretion was corroborated using *Rhodobacter sphaeroides* lipopolysaccharide (Rs-LPS), which is a competitive TLR4 inhibitor that does not produce TLR4 activation [48,49]. Rs-LPS was highly effective at inhibiting TNFα secretion from microglia (Figure 5C).

CD14 is required for TLR4 endocytosis and downstream signaling [50]. Anti-CD14 antibody significantly impairs TLR4 signaling [51] and suppresses LPS-induced TNFα secretion [52,53,54,55]. Pretreatment of microglia with anti-CD14 antibody significantly reduced metHgb-induced TNFα secretion (Figure 5C).

**Figure 6:**
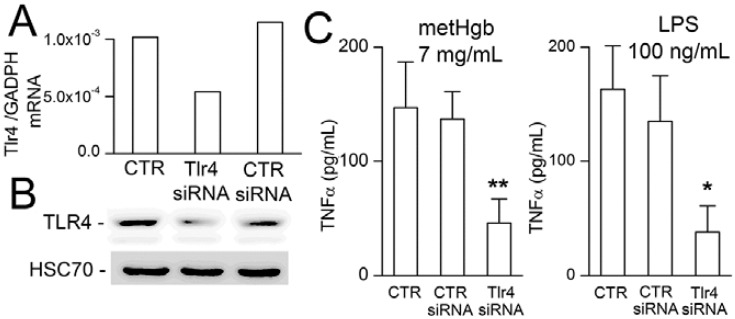
Suppression of *Tlr4* reduces metHgb-induced TNFα secretion in microglia. (**A**,**B**) *Tlr4* mRNA measured by qPCR (**A**) and TLR4 protein measured by immunoblot (**B**) under control conditions (CTR), and after transfection with control siRNA or siRNA directed against *Tlr4*, as indicated; mRNA and protein from 5 wells pooled for analysis; (**C**) TNFα secretion induced by metHgb (**left** panel) or LPS (**right** panel) under control conditions or with siRNA suppression of *Tlr4*, as indicated; 5 replicates per condition; * *p* < 0.05; ** *p* < 0.01.

To further establish the role of TLR4 in metHgb-induced TNFα secretion, microglia were transfected with siRNA directed against *Tlr4*. Gene suppression was evaluated by qPCR and immunoblot, which showed reductions in *Tlr4* mRNA and TLR4 protein of ~50% (Figure 6A,B). Gene suppression of *Tlr4* was associated with commensurate suppression of both metHgb- and LPS-induced TNFα secretion (Figure 6C).

LPS-free metHgb maintained in solution under anaerobic conditions at 37 °C for 7 days, which we showed maintained its integrity by FT-ICR mass spectrometry, also maintained its potency in causing TNFα secretion from microglia (Figure 5D).

### 2.5. metHgb Is the Main Constituent of Hemolysate Responsible for TLR4 Activation

Having shown that metHgb is a TLR4 ligand, we sought to determine whether other constituents of hemolysate, which were shown during our purification experiments (Figure 1, lanes 4, 5), might also activate TLR4. For this experiment, the last step alone in our purification procedure, the endotoxin removal chromatography column (EndoTrap HD), was applied to the commercial preparation of hemolysate, which is predominantly metHgb, to obtain LPS-free hemolysate. Comparing the effect on TNFα secretion of LPS-free hemolysate to that of purified LPS-free metHgb showed <2% greater efficacy of hemolysate (Figure 5E), consistent with metHgb accounting for >98% of the TLR4-activating efficacy present in hemolysate.

### 2.6. metHgb Induces Neuroinflammation

**Figure 7:**
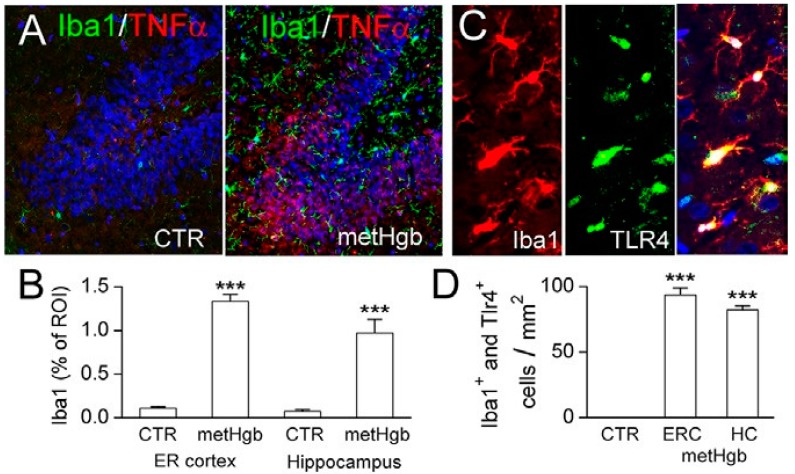
Infusion of highly purified LPS-free metHgb into the subarachnoid space of the entorhinal cortex induces robust neuroinflammation. (**A**) Immunolabelings for Iba1 (green) and TNFα (red) in hippocampus in control brain (**left** panel) and after metHgb infusion (**right** panel); (**B**) Quantification showed robust upregulation of Iba1 in both the entorhinal (ER) cortex and in the hippocampus, compared to controls (CTR); 3 rats per group; *** *p* < 0.001; (**C**) Immunolabelings for Iba1 (red) and TLR4 (green) in hippocampus after metHgb infusion; merged images also are shown (right panel); (**D**) Quantification showed marked increases in cells co-expressing Iba1 and TLR4 in the entorhinal cortex (ERC) and in the hippocampus (HC), compared to controls (CTR).

Purified LPS-free metHgb was infused into the subarachnoid space of the entorhinal cortex of rats [56,57]. Immunolabeling of brain sections for ionized calcium binding adaptor molecule 1 (Iba1) and TNFα showed robust microglial activation in the adjacent entorhinal cortex as well as remotely in the hippocampus (Figure 7A,B). Immunolabeling also showed that microglial activation was accompanied by microglial upregulation of TLR4 (Figure 7C,D).

### 2.7. Discussion

The principal findings of the present study are: (i) at physiologically relevant concentrations, highly-purified, LPS-free metHgb causes secretion of TNFα from microglial and macrophage cell lines; (ii) metHgb-induced secretion of TNFα is inhibited by *Tlr4* gene suppression as well as by highly specific TLR4 inhibitors; (iii) metHgb activates TLR4 in a CD14-dependent manner; (iv) metHgb infusion into the subarachnoid space *in vivo* causes microglial activation and upregulates TLR4 and TNFα.

Except for the well-documented role of endothelin in cerebral vasospasm [58,59], the molecular mechanisms responsible for most of the adverse aftereffects of SAH remain obscure. In SAH, mechanisms of injury are divided broadly into mechanisms of “early brain injury” (EBI) and mechanisms of “delayed brain injury” (DBI) [60,61,62,63]. EBI is linked to an abrupt rise in intracranial pressure that triggers transient global ischemia, whereas DBI is viewed as being due largely to the neurotoxic and neuroinflammatory effects of blood breakdown products. These broad categories encompass a variety of discrete molecular and cellular mechanisms, not necessarily mutually exclusive, that include oxidative stress, cerebral edema, inflammation, apoptosis, cortical spreading ischemia, vasospasm, microthrombosis and others. Our focus here specifically on TLR4-mediated neuroinflammation should not be taken to imply that we regard this mechanism, by itself, as being sufficient to account for all SAH-induced injury. It has long been held that SAH-induced injury is due not any single mechanism but likely to a combination of mechanisms, a concept that has been used to argue for a multifaceted approach to treatment using pleiotropic drugs [64].

As erythrocytes lyse after SAH, the brain is exposed to high concentrations of hemoglobin [65], which can induce injury via several distinct mechanisms. Hemoglobin, largely in the form of oxyHgb, has been linked to the induction of vasospasm following SAH, due in part to scavenging of nitric oxide [66]. metHgb can be oxidized to the proinflammatory ferrylHgb[Fe^4+^] by H2O2 released from macrophages or neutrophils [67,68]. The major degradation product of hemoglobin, iron, has been linked to important cellular changes including cell death—iron overload in the acute phase of SAH causes oxidative injury leading to neuronal cell death, with these effects ameliorated by the iron chelator, deferoxamine [69]. Hemoglobin-associated injury that is independent of TLR4, such as that due to oxyHgb, ferrylHgb and iron, is highly likely to contribute to the overall outcome after SAH.

Previous work with gene deletion showed convincingly that TLR4 plays a crucial role in SAH, specifically in vasospasm and neuronal death [24]. However, previous work left unanswered how TLR4 activation occurs. Three molecules derived from erythrocyte breakdown have been postulated to be endogenous TLR4 ligands: metHgb [28,29], heme [30,31] and hemin [32], whereas other breakdown products, including oxyHgb [35], ferrylHgb [67,68] and non-heme porphyrins [30] are reported not to be TLR4 ligands. Poor water solubility of heme and hemin, and potential contamination of metHgb by LPS confounded our previous understanding of the potential role of hemolysate products as endogenous TLR4 ligands in SAH. Many other injury-associated molecules have been identified as endogenous TLR4 ligands [15], but a component of blood, and specifically an erythrocyte breakdown product, rather than a plasma constituent, is generally held to be responsible for SAH-related injury [27].

Our principle goal was to establish that metHgb ligation of TLR4 is an important mechanism that contributes to SAH-induced injury. Our data establish that metHgb is a TLR4 ligand at physiologically relevant concentrations, and that metHgb accounts for >98% of the TLR4-activating efficacy present in LPS-free hemolysate. In a recent review of 23 reported endogenous ligands of TLR2 and/or TLR4, fully 8 were shown to have no cytokine stimulatory effects when highly purified preparations were used [15], underscoring the critical importance of testing highly purified LPS-free compounds. Here, we used a 5-step process to obtain highly purified LPS-free metHgb, and we confirmed its purity and absence of LPS using FT-ICR mass spectrometry and the LAL assay. Notably, the recent review of endogenous ligands [15] did not include metHgb, heme or hemin among endogenous ligands, presumably because the TLR4 activity of these compounds had not been properly established with purified LPS-free compounds. The work reported here confirms that metHgb now should be included among endogenous TLR4 ligands.

The classic TLR4 ligand LPS previously was shown to induce TNFα secretion in macrophages and microglia [44,45,46], as was LPS-contaminated metHgb in macrophages studied in the presence of polymyxin B [28,29]. Here, using purified LPS-free metHgb, we showed that this endogenous TLR4 ligand induces secretion of TNFα from microglia and macrophage cell lines. In addition, we showed that, when infused into the subarachnoid space, purified LPS-free metHgb causes microglial activation and upregulation of TLR4 and TNFα. Importantly, we found that purified LPS-free metHgb maintained its potency when stored under anaerobic conditions at 37 °C for 1 week, a finding that will facilitate future studies on animal models of SAH.

Although previous work with gene deletion showed that TLR4 plays a crucial role in vasospasm and neuronal death [24], it remains to be determined whether TLR4 also plays a role in cognitive dysfunction or brain atrophy. Circumstantial evidence, however, suggests that it might. Persistent activation of TLR4 in the brain is one of the few molecular mechanisms established as a cause of profound and long-lasting cognitive dysfunction [16,17,18], with cognitive dysfunction being TNFα-dependent [19,20]. In addition, brain atrophy has been previously linked to persistent inflammation [10,11,12,13]. Future work will be required to determine the role, if any, of TLR4-mediated neuroinflammation in SAH-induced cognitive dysfunction and brain atrophy.

## 3. Experimental Section

### 3.1. Purification of Bovine metHgb

Endotoxin-free, cell culture-grade buffers, media, and reagents were used. Purified Hgb was obtained via tangential flow filtration [70]. Purified metHgb was prepared from lyophilized bovine hemolysate (“Hemoglobin from bovine blood”; Sigma-Aldrich, St. Louis, MO, USA). Because Hgb is readily oxidized in air, this commercial preparation of hemolysate is predominantly metHgb[Fe^3+^]. About 2 g of hemolysate powder was dissolved into 20 mM Tris-HCl buffer (pH 7.5) to make a final concentration of 100 mg/mL. Insoluble materials were removed by centrifugation at 15,000× *g* for 10 min at 4 °C. The hemolysate solution was applied to a Q-Sepharose fast flow column (GE Healthcare Life Sciences, Piscataway, NJ, USA), which was equilibrated with 20 mM Tris-HCl buffer, and the flow-through fraction was collected. Bound proteins were eluted by stepwise elution with 0.1 and 0.5 M NaCl for further analysis. Most of the metHgb was detected in the flow-through fraction. The flow-through fraction was concentrated up to 2-fold by a commercial ultrafiltration unit, Amicon Ultra-4 Centrifugal Filter with nominal molecular weight limit of 30 kDa (EMD Millipore, Billerica, MA, USA) following the manufacturer’s instructions. To remove contaminants of small molecular mass such as free heme, the concentrated metHgb solution from the ultrafiltration unit was applied to a desalting gel filtration column equilibrated with saline, with an exclusion limit of 5 kDa (PD-10; GE Healthcare Life Sciences). To remove any potential contamination by LPS, the metHgb solution from the PD-10 desalting column was then applied to an endotoxin removal chromatography column, EndoTrap HD (Hyglos GmbH, Starnberger See, Germany) [35]. Protein concentrations were determined by a protein assay kit (Bio-Rad, Hercules, CA, USA) using the initial hemolysate solution as a reference. Purity, abundance, and identity of the purified metHgb were validated by SDS-PAGE with Coomassie G-250 staining (Life Technology, Grand Island, NY, USA), immunoblot analysis with hemoglobin α subunit antibody (Santa Cruz Biotechnology, Dallas, TX, USA), and mass spectrometry. The absence of detectable LPS in the purified metHgb sample was confirmed using the LAL assay (Thermo Fisher Scientific, Rockford, IL, USA).

### 3.2. Mass Spectrometry

The molecular mass of the purified metHgb was measured using the Bruker 12T Solarix XR Fourier Transform Ion Cyclotron Resonance (FT-ICR) mass spectrometer (Bruker Daltonics, Bremen, Germany) at the University of Maryland Baltimore, School of Pharmacy Mass Spectrometry Center. All of the solvents used for mass spectrometry were HPLC grade. Briefly, samples for mass spectrometry were prepared by mixing 105 µg of purified metHgb with 200 µL of a mixture of water/methanol/formic acid (50:50:0.1 by volume). The prepared sample was infused at 3 μL/min, and a 4 kV spray voltage was applied for electrospray ionization. To mitigate the effect of instantaneous signal fluctuations, we obtained the final mass spectra by averaging data from 1000 scans. The molecular mass of the sample was determined by deconvoluting the mass spectra data using commercial software (Bruker Data Analysis 4.2, Bruker, Bremen, Germany).

### 3.3. Cell Culture

The N9 microglial cell line (Neuro-Zone, Milan, Italy) was maintained in Iscove’s Modified Dulbecco’s Medium (Life Technologies, Grand Island, NY, USA) with 5% fetal bovine serum (FBS) and antibiotics (100 units/mL penicillin and 100 μg/mL streptomycin) at 37 °C and 5% CO2. The J 774.2 macrophage cell line (Sigma Aldrich) was maintained in Dulbecco’s modified eagle medium (Life Technologies with 5% heat-inactivated FBS and antibiotics (100 units/mL penicillin and 100 μg/mL streptomycin) at 37 °C and 5% CO2. For ELISA and immunofluorescence experiments, 75,000 cells were seeded in 24 well culture dishes or on uncoated glass coverslips, respectively, and allowed to adhere overnight prior to exchanging media for experimental conditions.

### 3.4. TNFα ELISA

The N9 microglial cells and J 774.2 macrophage cells were exposed to the experimental conditions in 500 µL culture medium, and supernatants were collected for TNFα measurements. Immunoreactive TNFα levels were determined using a mouse TNFα Quantikine ELISA kit (R&D Systems, Minneapolis, MN, USA). All procedures were performed according to the manufacturer’s instructions.

### 3.5. siRNA Transfection

For experiments with siRNA, mouse *Tlr4* siRNA and nonspecific control siRNA were obtained from Santa Cruz Biotechnology. N9 microglial cells were cultured in 24-well culture dishes for 16 h and transfected with 100 nM siRNA using HiPerFect transfection reagent (Qiagen, Valencia, CA, USA). After 72 h, the N9 microglial cells were treated with LPS or metHgb for 24 h.

Gene suppression was checked by qPCR. Total RNA was extracted using Trizol Reagent (Invitrogen, Eugene, OR, USA), and the concentration of total RNA was determined by measuring the optical density at 260 and 280 nm. To avoid contamination by genomic DNA, the RNA was further treated with Amplification Grade DNase I (Invitrogen). cDNA was synthesized from 1 μg of total RNA of each sample using SuperScript III Reverse Transcriptase (Invitrogen). The abundance of *Tlr4* mRNA in the samples was determined by real-time PCR (ABI PRISM 7300; Applied Biosystems, Carlsbad, CA, USA). The abundance of *GAPDH* mRNA was measured to normalize the samples. The primers used were 5'-AGCTTCTCCAATTTTTCAGAACTTC-3' (forward) and 5'-TGAGAGGTGGTGTAAGCCATGC-3' (reverse) for *Tlr4*; 5'-CATCACTGCCACCCAGAAGACTG-3' (forward) and 5'-ATGCCAGTGAGCTTCCCGTTCAG-3' (reverse) for *GAPDH*.

Gene suppression also was checked by immunoblot. Cells were washed with cold PBS and lysed in cold lysis buffer (1.5 mM KH2PO4, 8 mM Na2HPO4 (pH 7.3), 3 mM KCl, 137 mM NaCl, and 1% Triton X-100) with protease inhibitor (Roche Applied Science, Indianapolis, IN, USA). Lysates were separated on NuPAGE BisTris Gels (Invitrogen), transferred onto PVDF membrane and analyzed by immunoblotting with antibodies against TLR4 (Santa Cruz Biotechnology). Bands were detected with the enhanced chemoluminescence detection method (Pierce, Rockford, IL, USA).

### 3.6. In Vivo metHgb Infusion

Brain infusions of metHgb were carried out as described previously from this lab [56,57], except that the injection of autologous blood was replaced by an injection of 150 µL purified LPS-free metHgb (140 mg/mL). The brains were examined at 24 h after the infusions. 

### 3.7. Immunohistochemistry

After transcardiac perfusion/fixation with 10% neutral buffered formalin, the brains were cryoprotected with 30% sucrose. Cryosections (10 μm) were blocked (5% goat serum, Sigma, plus 0.2% Triton X-100 for 1 h at room temperature) and then incubated overnight at 4 °C with the following primary antibodies: rabbit anti-Iba1 (Wako Pure Chemical Industries, Osaka, Japan) plus goat anti-TNFα (Santa Cruz Biotechnology); or goat anti-Iba1 (Abcam, Cambridge, MA, USA) plus rabbit anti-TLR4 (Santa Cruz Biotechnology).

After several rinses in phosphate buffered saline, the slides were incubated for 1 h with fluorescent-labeled species appropriate secondary antibodies (Alexa Flour 488 and Alexa Flour 555; Invitrogen) at room temperature. Omission of primary antibody was used as a negative control. The sections were coverslipped with polar mounting medium containing antifade reagent and 4',6-diamidino-2-phenylindole (DAPI; Invitrogen), and were examined using epifluorescence microscopy (Nikon Instruments Inc., Melville, NY, USA).

### 3.8. Statistical Analysis

Data are presented as mean ± standard error. Statistical comparisons were made using Student’s *t*-test or ANOVA, as appropriate, with post-hoc comparisons made using Fisher’s method. Calculations were performed with OriginPro8 (OriginLab Corp., Northampton, MA, USA). *p* < 0.05 was considered to be statistically significant.

## 4. Conclusions

We show that metHgb is an effective TLR4 ligand, that it can induce TLR4-dependent secretion of TNFα from microglial and macrophage cell lines, and that it can induce neuroinflammation when infused into the subarachnoid space in rats. Future work will be needed to determine the role of metHgb-induced TLR4 activation *in vivo* in SAH-induced cognitive dysfunction and brain atrophy.
